# Correction: ‘You’ve got dry macular degeneration, end of story’: a qualitative study into the experience of living with non-neovascular age-related macular degeneration

**DOI:** 10.1038/s41433-019-0559-z

**Published:** 2019-09-05

**Authors:** Deanna J. Taylor, Lee Jones, Alison M. Binns, David P. Crabb

**Affiliations:** Division of Optometry and Visual Science, School of Health Sciences, City, University of London, Northampton Square, London, EC1V 0HB UK

**Keywords:** Macular degeneration, Biological sciences

**Correction to**: **Eye**

10.1038/s41433-019-0445-8,

Published online 22 May 2019

This Article was originally published under the License © The Royal College of Ophthalmologists 2019, but has now been made available under a CC-BY 4.0 license. The PDF and HTML versions have been modified accordingly.

